# The initial engraftment of tumor cells is critical for the future growth pattern: a mathematical study based on simulations and animal experiments

**DOI:** 10.1186/s12885-020-07015-9

**Published:** 2020-06-05

**Authors:** Bertin Hoffmann, Tobias Lange, Vera Labitzky, Kristoffer Riecken, Andreas Wree, Udo Schumacher, Gero Wedemann

**Affiliations:** 1 Competence Center Bioinformatics, Institute for Applied Computer Science, University of Applied Sciences Stralsund, Zur Schwedenschanze 15, 18435 Stralsund, Germany; 2 Institute for Anatomy and Experimental Morphology, University Cancer Center, University Medical Center Hamburg-Eppendorf, Martinistraße 52, 20246 Hamburg, Germany; 3 Research Department Cell and Gene Therapy, Department of Stem Cell Transplantation, University Medical Center Hamburg-Eppendorf, Martinistraße 52, 20246 Hamburg, Germany; 4 Institute of Anatomy, Rostock University Medical Center, Gertrudenstraße 9, 18057 Rostock, Germany

**Keywords:** Tumor growth, Nonlinear systems, Parameter estimation, Nonlinear least squares, Gompertz model, Exponential model, Power model, Logistic model, Replacement, reduction and refinement (3Rs), Animal welfare

## Abstract

**Background:**

Xenograft mouse tumor models are used to study mechanisms of tumor growth and metastasis formation and to investigate the efficacy of different therapeutic interventions. After injection the engrafted cells form a local tumor nodule. Following an initial lag period of several days, the size of the tumor is measured periodically throughout the experiment using calipers. This method of determining tumor size is error prone because the measurement is two-dimensional (calipers do not measure tumor depth). Primary tumor growth can be described mathematically by suitable growth functions, the choice of which is not always obvious. Growth parameters provide information on tumor growth and are determined by applying nonlinear curve fitting.

**Methods:**

We used self-generated synthetic data including random measurement errors to research the accuracy of parameter estimation based on caliper measured tumor data. Fit metrics were investigated to identify the most appropriate growth function for a given synthetic dataset. We studied the effects of measuring tumor size at different frequencies on the accuracy and precision of the estimated parameters. For curve fitting with fixed initial tumor volume, we varied this fixed initial volume during the fitting process to investigate the effect on the resulting estimated parameters. We determined the number of surviving engrafted tumor cells after injection using ex vivo bioluminescence imaging, to demonstrate the effect on experiments of incorrect assumptions about the initial tumor volume.

**Results:**

To select a suitable growth function, measurement data from at least 15 animals should be considered. Tumor volume should be measured at least every three days to estimate accurate growth parameters. Daily measurement of the tumor volume is the most accurate way to improve long-term predictability of tumor growth. The initial tumor volume needs to have a fixed value in order to achieve meaningful results. An incorrect value for the initial tumor volume leads to large deviations in the resulting growth parameters.

**Conclusions:**

The actual number of cancer cells engrafting directly after subcutaneous injection is critical for future tumor growth and distinctly influences the parameters for tumor growth determined by curve fitting.

## Background

Experiments with mouse models are some of the most important experimental tools to study the growth of tumors, their metastatic progression and the effect of therapeutic interventions [1, 2]. In these types of experiments the animals are assigned to different groups, which are then subject to treatment or serve as control groups. Cells, often from commercially available human cell lines, are engrafted under the skin of genetically identical immunodeficient mice. After a period of time when the primary tumors are allowed to grow, the size of each tumor is normally measured by caliper every two or three days. At the end of the experiment, the animals are sacrificed and the primary tumors are excised and analyzed directly. Typically, the variations between groups in the mean values of primary tumor size measured on different days are considered to be indicators of differences in, for example, the effectiveness of a drug treatment [3–6]. Sometimes data from these experiments are fitted to mathematical growth functions to describe and predict the growth of the tumor [7–9]. This experimental procedure promises well-defined reproducible results and generates new insights into metastasis formation when mathematical modeling is applied [10]. However, despite the identical setup and careful execution of the experiments, the final size of the primary tumors typically varies by a factor of five [11]. The reasons for this wide range in size are not fully understood. We may assume that there is a large variation in the number of malignant cells that survive the first days following engraftment. Another possible effect may be variation in the growth parameters caused by, for example, slight differences in location of the engrafted cells affecting their proximity to the nearest blood vessels. It is therefore questionable whether the common approach of only using the mean values of primary tumor size measured on different days can deliver valid results, or whether the observed differences in fact represent random variations of the number of engrafted cells. In order to avoid this problem, data from each mouse can be fitted individually to mathematical functions such as the Gompertz growth function [12–14]. The resulting parameters of the mathematical functions provide meaningful information about the growth behavior of the tumor and differences between the different in vivo tumor models. These data are also important for the parametrization of mathematical and computer models of cancer growth and the influence of experimental therapeutic intervention on tumor growth and metastasis formation [15–18]. A systematic evaluation of whether this approach is delivering reliably accurate results, and under which circumstances these can be obtained, is the subject of the research presented in this article.

Different growth functions are in use to model the growth of the primary tumor mathematically, most notably the Gompertz and exponential growth functions [7, 12–14, 19, 20]. The choice of which of these two growth functions to use is not obvious, since in many cases growth saturation of the primary tumor is not reached during the experiment. Experimental errors make this identification even more difficult. Besides the Gompertz and exponential growth functions, other mathematical models exist that describe growth behavior depending on the type of tumor [21, 22], for example the power law, von Bertalanffy [23], generalized logistic [19, 24] or dynamic carrying capacity model. The latter is based on time-dependent carrying capacity as a result of the process of angiogenesis [25]. It is of fundamental interest, accurately identifying the type of growth function that best describes the growth behavior. Due to animal welfare considerations (the Three Rs: Replacement, Reduction and Refinement), only a small number of mice can be investigated and experiments are terminated once the tumors have reached 1.2 cm^3^ in volume [26]. However, the number of animals that have to be studied to identify the most suitable growth function is an open question.

Experimental xenograft mouse data suffer from many sources of experimental inaccuracies. Measurement is influenced by the epidermis, adipose tissue and fur of mice themselves, as well as unsystematic errors made by the laboratory staff. Furthermore, tumor volume determination with a manual caliper influences the results, since only two dimensions of the tumor can be measured while neglecting the depth of the tumor. In addition to the maximum likelihood estimation (MLE) method, the most common approach to determining growth parameters from a population or individual data affected by measurement errors is a curve fit applying least-squares minimization [21, 27, 28], which is available out of the box in mathematical computational software such as MATLAB [29], Origin [30] or Prism [31]. However, this method tends to yield unrealistic values of initial tumor volume. As an example, in the analysis of experimental data where 10^6^ cells were engrafted into mice, the least-square fit delivered results of less than 100 surviving tumor cells, which is biologically implausible [32]. It could also be argued that an incorrectly chosen model for the parameter estimation process causes this behavior. A common way to address the problem of unrealistic values is to fix the initial cell number as the number of injected cells during the parameter estimation process to reduce the number of degrees of freedom, as in Benzekry et al. [21], for example. However, there is no experimental data concerning the number of tumor cells that survive engraftment. We hypothesize that the percentage of cells surviving after s.c. injection into immunodeficient mice most likely varies considerably depending on the used cell line and mouse strain. A fixed initial cell number is a mere assumption and the consequences for parameter estimation are unclear at present.

Our research addresses the question of how growth of the primary tumor can be quantified by applying mathematical models, and investigates the limitations of different methodological approaches. We used self-generated synthetic data including random measurement errors to mimic a typical experimental setup. Known parameters of growth behavior were compared with the results from the least-squares curve fitting approach. Different sample sizes were used to investigate how many animals are necessary to make a well-founded decision about the most suitable growth function for the underlying tumor data using goodness of fit criteria. The influence of the frequency of tumor volume measurement was investigated based on curve fitting with free and fixed initial volume conditions. Because we found that the choice of a fixed initial volume had a large effect on parameter estimation, a first proof-of-concept animal experiment using a well-established xenograft model was performed to determine the number of viable cells on days 1, 2, 4 and 8 after injection by making use of the bioluminescent properties of the used tumor cells. Only viable cells are able to metabolize luciferin by the genetic overexpression of luciferase and hence to emit photons that can be detected by bioluminescence imaging. By measuring the photon flux emitted from tumor cells taken from in vitro culture in parallel (standard row ranging from 10^6^ to 10^1^ cells), we could estimate the number of viable cells in the skin tumor nodules on days 1 to 8 after injection to improve the parameter estimation process.

## Methods

### Cell culture

The prostate cancer cell line PC-3 (obtained from ATCC®, CRL-1435™) was cultured in RPMI-1640 medium containing 10% heat inactivated fetal bovine serum, 100 U/ml penicillin and 100 μg/ml streptomycin (Gibco, Paisley, UK) under standard cell culture conditions (37 °C, 95% relative humidity, 5% CO_2_).

### Lentiviral transduction

PC-3 cells were prepared for bioluminescence imaging (BLI) by lentiviral transduction using a mixture of three vectors (LeGO-Luc2-iCer2-Puro+, LeGO-Luc2-iV2-Puro+ and LeGO-Luc2-iC2-Puro+), adjusted to equal titers. Using this approach, we achieved stable expression of fire fly luciferase from *Photinus pyralis* (Luc2, Promega), fluorescent proteins (Cerulean, Venus or mCherry, respectively) and a resistance to puromycin (puromycin N-acetyltransferase) in the PC-3 cells as described before [33]. These PC-3 Luc2/RGB cells were cultured in medium as described above, but supplemented with 500 ng/ml puromycin.

### Animal experiments for detection of surviving engrafted cells

At 10 to 12 weeks old, 8 male NSG (NOD.Cg-Prkdc^scid^ Il2rg^tm1Wjl^/SzJ; Jax, Stock 005557) mice were subcutaneously (s.c.) injected with 10^6^ PC-3 *Luc2/RGB* cells. On day 1, 2, 4 or 8 after tumor cell injection (*n* = 2) the NSG mice were anesthetized with xylazine/ketamine (120/16 mg/kg body weight, Bayer, Leverkusen, Germany / Graeub, Bern, Switzerland) and administered with luciferin (150 mg/kg body weight; Sigma, Steinheim, Germany) intraperitoneally, 10 min before sacrificing the mice (terminal cardiac blood collection with following cervical dislocation) for resection of s.c. tumor nodules. Mice were distributed randomly to the groups. The experiment was not blinded. In parallel, a cell standard of 10^6^ cells to one cell was plated in triplicate on a 96-well plate in 100 μl of 300 μg/ml D-luciferin potassium salt (Biosynth AG, Thal, Switzerland) diluted in Dulbecco’s phosphate-buffered saline (DPBS, Gibco, Paisley, UK). Tumors and prepared cells were measured in the in vivo imaging system (IVIS 200, Perkin Elmer, Waltham, MA, USA) for BLI signals. The signal intensity was analyzed by total flux (p/s/cm^3^/sr) with Living Image Software (Perkin Elmer, Waltham. MA, USA).

The animal experiments were approved by the local animal experiment approval committee (Behörde für Gesundheit und Verbraucherschutz, Amt für Verbraucherschutz, Lebensmittelsicherheit und Veterinärwesen, Freie und Hansestadt Hamburg, assigned project No. G80/16). They are in accordance with the relevant national and international guidelines. The animals were housed with a 12 h day-night cycle in a temperature- (21 °C) and humidity- (50%) controlled room. All mice were kept in individually ventilated cages under pathogen-free conditions, fed with sterile standard food and water ad libitum.

### Statistical analyses of animal experiments

Due to the proof-of-principle character of this animal experiment, a cohort size of *n* = 2 was used. Moreover, we aimed to analyze the dynamics in tumor cell survival early after injection. Therefore, we did not intend to perform statistical tests between different groups.

### Mathematical models

Mathematical models describe the change in tumor volume *V* over time *t*. We focused our study on four growth functions: exponential, Gompertz, power and generalized logistic. These functions are widely used to describe and predict the growth behavior of tumors [21, 22, 34–36]. In the presented models, growth starts from an initial tumor volume *V*_*0*_, which is the number of engrafted tumor cells at the beginning of the experiment.

#### Exponential

The simplest tumor growth behavior is exponential growth, where the cells divide regularly (constant doubling time) and growth is not decelerated by limitations of nutrients and space. This behavior is usually observed at the early stages of tumor development [20]. Exponential growth is defined by.

1 $$ V(t)={V}_0{e}^{at} $$

where *a* is the initial proliferation rate at *V*_*0*_.

#### Gompertz

The Gompertz function is sigmoid, with a characteristic “S”-shaped curve, and is the most common function used to describe tumor growth. In the initial stages the growth pattern corresponds to an exponential function. The tumor grows over time and requires more and more nutrients and space, which become less available with time. At a certain size, tumor growth reaches saturation and the curve levels off. The Gompertz growth is defined by.

2 $$ V(t)={V}_0{e}^{\frac{a}{\beta}\left(1-{e}^{-\beta t}\right)} $$

where *a* is the initial proliferation rate and *β* is the rate of exponential decay, which is controlled by environmental conditions.

#### Generalized logistic

The generalized logistic equation is another type of S-shaped curve that is limited by a carrying capacity denoted by *K.* The growth rate *a* decreases linearly in proportion to the carrying capacity [24]. Generalized logistic growth is defined [35] by.

3 $$ V(t)=\frac{K\kern0.5em {V}_0\kern0.5em {e}^{at}}{K+{V}_0\kern0.5em \left({e}^{at}-1\right)} $$

#### Power

A power function can also be used to describe growth behavior for some types of tumor [37]. The power function is defined [35] by.

4 $$ V(t)={\left({V}_0^{1-\alpha }+\left(1-\alpha \right) rt\right)}^{1/\left(1-\alpha \right)} $$

where *α* describes the fraction of the tumor that is able to grow (*α* < 1) and *r* is the initial proliferation rate at *V*_*0*_.

### Generating synthetic data

In order to evaluate parameter estimation, we generated synthetic data including measurement errors. These errors were quantified using experimental data by Benzekry et al. [21]. We used the statistical framework from this publication to generate synthetic tumor data *Y*, including errors, applying the following mathematical formalism:

5 $$ {Y}_i^j=M\left({t}_i^j,\beta \right)+{\sigma}^j{E}_i^j{\varepsilon}_i^j $$

This framework generates for animal *j* at time ${t}_i^j$, the deterministic volume ${y}_i^j=M\left({t}_i^j,\beta \right)$ based on a deterministic model *M*, which depends on a parameter vector *β*. Measurement errors are added to the deterministic volume ${y}_i^j$, where ${\varepsilon}_i^j\sim \mathcal{N}\left(0,1\right)$ is a random Gaussian variable and ${\sigma}^j{E}_i^j$ is the standard deviation of the error. The following error model was applied to generate individual measurement errors for each animal:

6 $$ {E}_i^j=\left\{\begin{array}{c}{\left({y}_i^j\right)}^{\alpha },{y}_i^j\ge {V}_m\\ {}{V}_m^{\alpha },{y}_i^j<{V}_m\end{array}\right\} $$

where *V*_*m*_ is the measurement threshold that represents the smallest measurable tumor volume using a caliper. The parameters *V*_*m*_ = 83 mm^3^, α = 0.84 and *σ* = 0.21 from the literature were used [21].

For ${y}_i^j$ eqs. (1) (2) (3) and (4) were used with the parameters displayed in Table 1. Parameters for Gompertz growth were taken from Benzekry et al. [21]. The growth parameters for all other growth functions were derived from the Gompertzian synthetic data. The growth parameters were manually tuned until the generated synthetic data showed a tumor volume of (1730 mm^3^ ± 130 mm^3^) as the Gompertz data at the end of the experiment. Parameter *V*_0_ was set to 1 mm^3^ for all growth functions. The generation of synthetic data was implemented with MATLAB (MATLAB R2019a, The MathWorks Inc., Natick, MA, USA).

**Table 1:** Summary of the selected parameters for the generation of synthetic data

Function	Parameter	Value
Exponential	*a* [day^− 1^]	0.175
Gompertz	*a* [day^−1^]	0.56 [21]
	*β* [day^−1^]	0.719 [21]
Generalized logistic	*a* [day^−1^]	0.19
	*K* [mm^3^]	3500
Power	*r* [day^−1^]	0.78
	*α*	2/3

### Curve fitting procedure

A common problem with the analysis of experimental data is the limited duration of experiments due to animal welfare considerations. Often this leads to an absence of data points in the advanced stages of the experiment where the tumor would have reached its maximum size, corresponding to the saturated area of a Gompertz or logistic function. To mimic a common experimental setup [38], we analyzed *N* data points ranging from days 23 to 43, where the number of data points *N* depends on the measuring frequency of 1, 2, 3 or 4 days between each measurement of the tumor volume.

Least-squares minimization was performed using *lsqcurvefit* (trust-region reflective algorithm) from the MATLAB Optimization Toolbox (MATLAB R2019a, The MathWorks Inc., Natick, USA) to obtain growth parameters based on *N* synthetic tumor volume data ${Y}_i^j=\left[{Y}_1^j,\dots, {Y}_N^j\right]$ for each individual dataset *j.* The fits were performed to obtain all parameters depending on the mathematical model *M* without the help of any prior knowledge, for example well-known growth rates or carrying capacities for specific cell lines. We also performed the same fits but with a fixed initial tumor volume *V*_0_ = 1 mm^3^, which is widely used to reduce the number of degrees of freedom [21, 28, 35, 39] (as examples among many other studies). For comparison, we performed the same fits with different initial tumor volumes *V*_0_ = {1.0, 0.8, 0.6, 0.4, 0.2, 0.1} to study the influence of an incorrectly assumed fixed initial tumor volume on the estimated growth parameters.

### Goodness of fit criteria

To determine which of the selected growth functions (1) to (4) best modeled the growth behavior of the synthetic data, different fit metrics were used. A classical goodness of fit criterion is the root mean squared error (RMSE), which sums up the deviations between the observed values *y*_*i*_ and the predicted values ${\hat{y}}_i$ (residuals):

7 $$ {RMSE}^j=\sqrt{\frac{1}{N}}{\sum}_{i=1}^N{\left({y}_i^j-{\hat{y}}_i^j\right)}^2 $$

The RMSE value can range from 0 to ∞ depending on the scale of the data (absolute measure of fit). Overall, the lowest RMSE value can indicate the best mathematical model to describe the underlying data.

Another criterion is the coefficient of determination denoted by R^2^ (R squared):

8 $$ {R}^{2j}=1-\frac{\sum_{i=1}^N{\left({y}_i^j-{\hat{y}}_i^j\right)}^2}{\sum_{i=1}^N{\left({y}_i^j-{\overline{y}}_i^j\right)}^2} $$

where $\overline{y}$ is the overall mean of the observed values. R^2^ describes how well the regression predictions approximate the observed data points. A value of R^2^ = 1 indicates that a chosen model perfectly fits the underlying data. Lower values of R^2^ indicate a non-perfect fit. Even negative values are possible, if the summed squared error based on the estimated fit curve is greater than the summed squared error based on the mean line.

To take into account the fact that different models have different numbers of degrees of freedom that can be fitted, the Akaike information criterion (AIC) [40, 41] was calculated, which penalizes a higher number of free growth parameters. AIC is defined by.

9 $$ {AIC}^j=N\ast \ln \left(\frac{\sum_{i=1}^n{\left({y}_i-{\hat{y}}_i\right)}^2}{N}\right)+2k $$

where *k* is the number of free parameters. When the number of data points *N* is small compared to the number of parameters *k*, a corrected version of AIC is more accurate:

10 $$ { AIC c}^j={AIC}^j+\frac{2k\left(k+1\right)}{N-k-1} $$

### Accuracy and precision

To investigate the influence of different measurement frequencies (1, 2, 3 or 4 days between each measurement), the parameter estimation bias (peb) [42] was calculated to assess the accuracy of each parameter:

11 $$ peb=\frac{\sum_{k=1}^n\left(\frac{\theta^{\ast }-\theta }{\theta}\right)}{n} $$

where *θ*^∗^ is the estimated parameter value, *θ* is the true parameter value of the synthetic data and *n* is the number of samples within the population. For example, a peb of ±0.10 represents a deviation of ±10% from the original parameter. To describe the precision (statistical variability) of the estimated parameters, the coefficient of variation was calculated:

12 $$ CV=\frac{\sigma }{\overline{x}}\ast 100 $$

where *σ* is the standard deviation of each parameter within the population and $\overline{x}$ is the mean value of each parameter.

## Results

### Applying nonlinear curve fitting with a fixed initial tumor volume improves the accuracy of the estimated parameters

Some time after subcutaneous injection of the tumor cells, the primary tumor at the injection site becomes large enough to be measured. The frequency of measuring the size of the tumor depends on various criteria, such as the availability of qualified staff, the goal of the experiment and the regulations at the research facility.

**Fig. 1:**
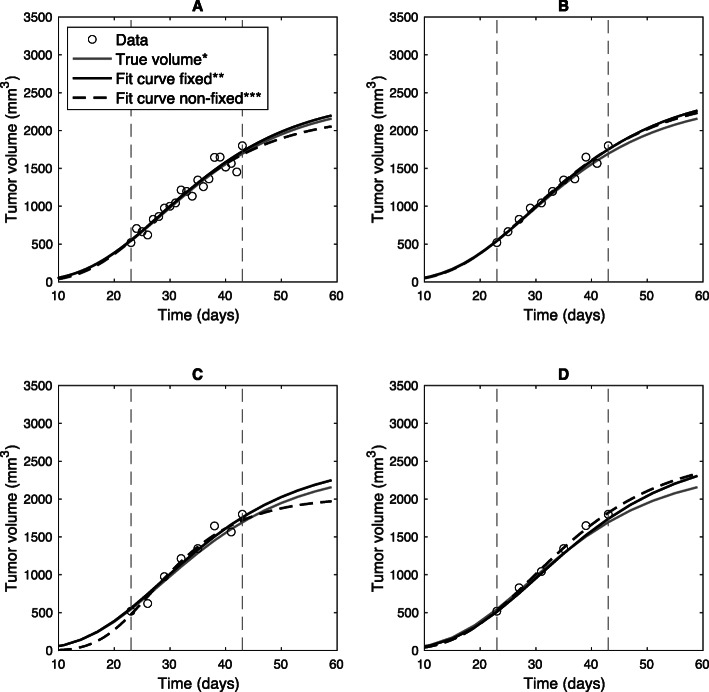
Example of estimated growth curves based on different data availability. Representative example of estimated growth curves and their forecast performance. Each fit was performed based on the same synthetic sample, but different data points from that sample set were used. *True synthetic tumor growth without errors. Fit curves based on different number of days between each data point: **a** = 1, **b** = 2, **c** = 3 and **d** = 4 days. The initial tumor volume *V*_0_ was fixed to 1 mm^3^ (**) and non-fixed (***) during curve fitting

Gompertzian based synthetic data were evaluated by applying nonlinear curve fitting under conditions of fixed (*V*_0_ = 1 mm^3^) and non-fixed initial tumor volume to compare the accuracy and precision of the estimated parameters. Fixing the initial tumor volume to a constant value is believed to be more precise due to the reduced number of degrees of freedom [21, 28, 35, 39]. As an example of using a fixed vs. non-fixed initial volume during the fitting procedure, Fig. 1 shows growth curves of fits on a single synthetic sample with different measurement frequencies compared to the true volume which represent the synthetic tumor growth with the true set of growth parameters without measurement errors. The growth curves with a fixed initial tumor volume look similar ($\overline{RMSE}=94.19\pm 2.96$) in the period under consideration (day 23 to day 43, gray dashed lines), but there are differences in the further course of the tumor growth curve ($\overline{RMSE}\ 214.59\pm 16.75$). However, the predictions in Fig. 1 are still within one and two standard deviations of the measurement error for the fixed and non-fixed fit curve, respectively. In order to check the accuracy and precision of the estimated parameters numerically, the parameter estimation bias (peb) and the coefficient of variation (CV) were calculated for a sample of 20 animals (Table 2). Additionally, we investigated another period under consideration (day 14 to day 43) to include more tumor volume data points beginning from ~ 150 mm^3^ (Additional file 2). This data support our findings for data points beginning from ~ 500 mm^3^. In general, the precision and accuracy decrease with decreasing measuring frequency, especially when *V*_0_ is not fixed during the fitting procedure. We conclude that the best results in terms of parameter estimation can be obtained with a one-day measuring frequency and a fixed initial tumor volume. However, a measuring frequency of three days is also sufficient to describe the underlying data points, since the determined parameters deviate from the correct value by less than 2% (peb =  ± 0.02) in combination with a fixed initial tumor volume (Table 2). Furthermore, with an increasing number of data points the estimated parameters will be more accurate, especially with a non-fixed initial tumor volume (see Additional file 2). Despite these results, we do not recommend performing curve fitting without a fixed initial tumor volume.

**Table 2:** Parameter estimation of 20 synthetic samples of Gompertzian growth based on different measurement frequencies

Parameter	Estimated values^**1**^	Estimated values^**3**^	Peb^**1**^	Peb^**3**^	CV^**1**^	CV^**3**^
*V*_0_ [mm^3^]	3.61 (0.01–9.48)	5.11 (0–27.48)	2.6141	4.1113	86.8475	164.8952
*a* [day^−1^]	0.52 (0.3–1.19)	0.69 (0.2–1.55)	−0.0788	0.2290	44.2930	57.3469
*β* [day^−1^]	0.0664 (0.0524–0.0952)	0.0716 (0.0376–0.1031)	−0.0765	−0.0035	18.7014	27.685
*a*^a^ [day^−1^]	0.55 (0.54–0.58)	0.55 (0.52–0.59)	−0.0104	−0.0156	2.0529	3.1901
*β*^a^ [day^−1^]	0.0709 (0.0681–0.0758)	0.0702 (0.0655–0.0769)	−0.0143	−0.0231	2.6240	4.0617

The mean values for each parameter are indicated. The minimum and maximum values for each parameter are given in parentheses. Estimated parameters and fit metrics are based on different measurement frequencies of one^1^ and three^3^ days between each measurement of the tumor volume. For example, the parameter estimation bias peb^1^ and peb^3^ show results based on measurement intervals of one day and three days, respectively

^a^Parameter *V*_0_ was set to 1 mm^3^ during the fitting procedure

True values: *V*_0_ = 1 mm^3^, *a* = 0.56 day^− 1^, *β* = 0.0719 day^− 1^. A full version of this table is given in Additional file 1

### Measuring tumor size every day improves long-term predictability of growth model

Inspired by the results of parameter estimation with fixed and non-fixed initial tumor volumes, we evaluated Gompertzian synthetic data by applying nonlinear curve fitting with a fixed (*V*_0_ = 1 mm^3^) initial tumor volume to investigate the effect of the frequency of tumor size measurement on long-term predictability. To predict further tumor growth, the deviation between the true tumor volume ${y}_T^j$ and the estimated tumor volume ${\hat{y}}_i^j$ was calculated for each sample *j* at time *t*^*j*^ + *d*, where d is the prediction depth (Table 3). The predictive power decreased with decreasing measurement frequency and increased depth. Statistical tests of differences between measuring frequencies were performed based on the quadratic error of the deviation. There is a significant difference (*p* < 0.05) between a measuring frequency of 1 day and the others but no significant difference between frequencies 2 and 3 days (*p* > 0.05). This behavior was observed for all studied prediction depths. In order to evaluate the predictive power for different growth behaviors, we investigated a slower (Additional file 3) and faster (Additional file 4) growth behavior. There is a significant difference (*p* < 0.05) between a measuring frequency of 1 day and the others, but no significant difference (*p* > 0.05) between the measuring frequencies 2, 3 and 4 days for the slower growth behavior. No significant difference (*p* > 0.05) between measurement frequencies of 1, 2 and 3 days could be found, but a measurement frequency of 4 days is significant different (*p* < 0.05) compared to the other ones for the fast growth behavior. We assume that the observed difference in prediction is due to the different availability of data points: In the case of fast growth, there are more data points on the saturation region of the Gompertz function in the period under consideration, which makes the prediction of growth more accurate. Overall, we concluded that the best results in terms of tumor growth prediction were obtained with a one-day measurement frequency. Therefore, we recommend daily measurement in combination with a fixed initial tumor volume to obtain a good prediction of further tumor growth.

**Table 3:** Deviation between true and predicted tumor volumes using different time intervals between measurements

Measuring frequency (days between measurements)	$\overline{\boldsymbol{RMSE}}$	Absolute mean deviation from true volume at depth d [mm^**3**^]			
		1	3	5	10
1	79.00	28.37 (16.64; 40.1)	33.76 (20.15; 47.37)	38.97 (23.4; 54.54)	51.03 (30.67; 71.39)
2	84.06	57.22 (39.19; 75.25)	66.54 (45.92; 87.16)	76.49 (53.9; 99.08)	99.28 (72.07; 126.49)
3	83.94	49.8 (37.8; 61.8)	59.04 (45.27; 72.81)	68.01 (52.04; 83.98)	91.18 (71.16; 111.2)
4	90.79	84.99 (59.68; 110.3)	98.42 (68.92; 127.92)	111.56 (77.93; 145.19)	143.3 (100.89; 185.71)

The absolute mean deviation values based on 20 synthetic samples of Gompertzian growth. Indicated are the absolute mean values for each measurement frequency and the corresponding depth

() = 95% confidence interval. Parameter *V*_0_ was set to 1 mm^3^ during the fitting procedure. The mean RMSE was calculated based on the model fit in the period under consideration from day 23 to day 43

### The influence of sample size on the selection of a suitable growth function

An important question for planning animal experiments is the selection of group size to achieve reliable results. If the selected number of animals is too small, significant differences, for example in the effects of different drugs, cannot be interpreted correctly. This leads to unnecessary animal suffering and waste of resources. On the other hand, the number of animals should be as small as possible for animal welfare considerations.

We evaluated the influence of different sample sizes (number of animals) on the selection of a growth function for the underlying data using fit metrics for 10, 12, 15, 20 and 25 generated synthetic samples. Ten sets of samples were generated for each sample size and for each different growth function (Gompertz, exponential, power and logistic), as with only one randomly generated dataset there would be a risk of accidently drawing a non-representative sample. All growth parameters were determined by applying curve fitting with a fixed initial tumor volume of *V*_0_ = 1 mm^3^ and a measurement frequency of three days.

**Fig. 2:**
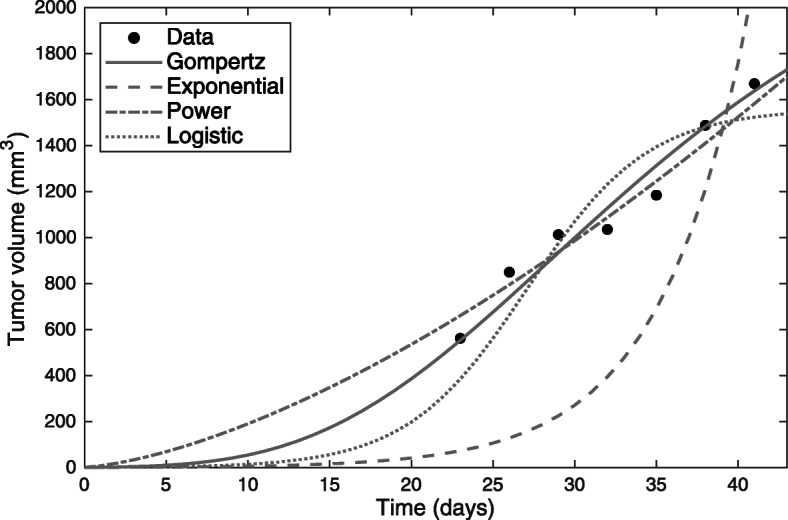
Estimated growth curves. Estimated growth curves based on a single synthetic Gompertz growth sample for all selected growth functions. The initial tumor volume *V*_0_ was fixed to 1 mm^3^ during curve fitting. Just from visual examination, the exponential growth function is not suited to describing the sample tumor data. Fit metrics can be used to compare the suitability of the other functions

As an example of the evaluation procedure, Fig. 2 shows a graphical representation of the estimated growth curves of a single synthetic sample based on Gompertzian growth behavior (see Table 1) including measurement errors. The calculated fit metrics RMSE, AIC, AICc and R^2^ for this sample are displayed in Table 4 (Fit metrics of all datasets based on Gompertzian growth are shown in Additional file 5). In these cases, the Gompertz function had the lowest RMSE and AIC/AICc value as well as the highest R^2^ value of all four tested growth functions. In nearly all cases AIC, RMSE and R^2^ led to the same decision regarding the most appropriate growth model (see Availability of data and materials). In subsequent analysis, we used only the RMSE value as a selection criterion for a specific growth function.

**Table 4:** Fit performance of four different growth models based on a single synthetic Gompertz dataset

Model	AIC	AICc	RMSE	R^**2**^	Number of parameters
Gompertz	61.22	64.22	59.57	0.97	2
Power law	65.28	68.28	79.62	0.95	2
Logistic	68.06	71.06	97.12	0.93	2
Exponential	93.37	96.37	592.01	−1.51	1

Models are ranked from the lowest to the highest RMSE value

**Fig. 3:**
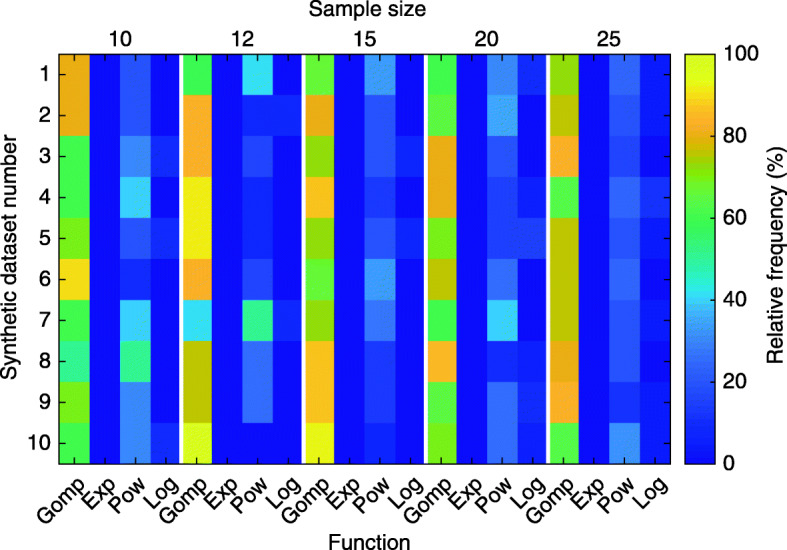
Effect of sample size on selection of most appropriate growth function for the underlying data. Each sample size (number of animals) was generated 10 times (synthetic dataset number) to mimic a Gompertzian growth behavior including measurement errors. For each growth function, Gompertz (Gomp), exponential (Exp), power (Pow) and logistic (Log), the RMSE value was determined for each individual sample within each dataset (e.g. dataset 2 and sample size 10). The relative frequency denotes how often each of the growth functions had the lowest RMSE value for an individual fit in each sample size group

Figure 3 shows the effect of different sample sizes on the selection of a growth function based on the RMSE value of the fit of the different growth functions to synthetic data based on Gompertz growth. In Additional files 6–8 the synthetic data is based on exponential, power and logistic growth. The relative frequency denotes how often one of the growth functions had the lowest RMSE value for an individual fit *j* in each sample size group. The difference in the RMSE value for two functions may be very small (< 1%), but the function with the lowest value will always be selected. A relative frequency of more than 50% for one of the growth functions indicates the most suitable function to model the underlying data.

The results show that 10 or 12 animals were not always sufficient to select the true Gompertzian growth behavior (Fig. 3: sample size 10, synthetic dataset #8; sample size 12, synthetic dataset #7). Similar results were also obtained for the power (Additional file 7: sample size 10, synthetic dataset #8; sample size 12, synthetic dataset #7) and logistic functions (Additional file 8: sample size 10, synthetic dataset #5). With a sample size of 15 animals, a relative frequency of over 50% was achieved for the correct growth function in each synthetic dataset (Fig. 3). Therefore, we recommend a minimum sample size of 15 animals to select a suitable growth function that can compensate for the measurement inaccuracies that occur when using calipers to measure tumor size every three days. Similar results were obtained when the evaluation was based on a measurement frequency of one, two or four days (see Availability of data and materials).

For synthetic data based on the exponential growth function, the procedure could not correctly identify the exponential growth function as the best suited function. As the synthetic data did not provide sufficient information about the saturated range, the Gompertz or power growth function was selected erroneously (Additional file 6).

### The influence of a fixed initial tumor volume on the estimated parameters

In a typical experimental setup, 10^6^ cells are subcutaneously injected into the mice to form a local tumor nodule. The tumor volume is measured over time and the growth behavior is analyzed by nonlinear curve fitting. In order to reduce the number of degrees of freedom the initial tumor volume is set to 1 mm^3^, which leads to more accurate parameter results during the curve fitting procedure (see Table 2). However, the assumption that all injected cells survive is questionable. There is no available data concerning the surviving cell numbers after injection. We therefore investigated the consequences of an incorrectly assumed initial tumor volume *V*_0_ during the curve fitting procedure for the determination of tumor growth.

**Fig. 4:**
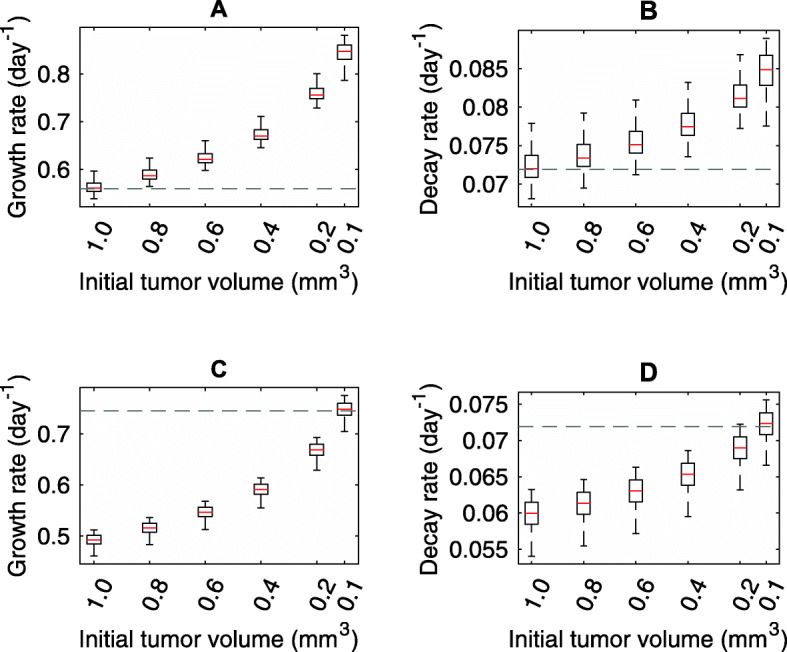
Box-plots of parameter estimation results using different initial volume conditions. The initial tumor volume was fixed at *V*_0_ = 1 mm^3^ (Panels **a** and **b**) and *V*_0_ = 0.1 mm^3^ (Panels **c** and **d**) (x-axis) during the process of fitting to a Gompertzian growth function. The resulting growth rates and decay rates are based on fits of 20 synthetic data samples. The gray dashed line marks the correct value

Figure 4 shows the deviation of the results for the growth rate *a* (Fig. 4ac) and decay rate *β* (Fig. 4bd) at different fixed initial tumor volumes during the fitting procedure, with a measuring frequency of one day and based on synthetic data with a true initial tumor volume of ${V}_0^t$ = 1 mm^3^ (Fig. 4ab) and ${V}_0^t$ = 0.1 mm^3^ (Fig. 4cd). (See Additional files 9 and 10 for measuring frequencies of two, three and four days based on true tumor volumes of ${V}_0^t$ = 1 mm^3^ and ${V}_0^t$ = 0.1 mm^3^, respectively). As 10^6^ cells were injected into the mice, we only chose cell numbers less than 10^6^ cells for the evaluation. Individual fits were performed based on 20 synthetic Gompertz growth samples. Incorrectly assumed values for ${V}_0^a$ led to large deviations from the correct parameters (Fig. 4). Consequently, an incorrectly estimated growth rate could lead to misinterpretation of the aggressiveness of the tumor.

In many cases no significant differences of the predicted tumor growth was found for two different assumed initial tumor volumes ${V}_0^a$ = 1 and ${V}_0^a$ = 0.1 and true initial volume ${V}_0^t$ = 1 (Table 3 and Additional file 11) and ${V}_0^t$ = 0.1 (Additional files 12 and 13) with regards to Fig. 4 ab and cd, respectively. As we have already described, the accuracy of the prediction depends not only on the estimated parameters, but also on the growth behavior in the period under consideration. For example, in the case of a fast growth behavior, there are more data points in the saturation phase of a Gompertzian growth, which improves the quality of the fit obviously. Therefore, a free selection of the initial volume of the tumor (in the range of biological plausible values) can have a negative effect on the quality of the prediction.

### The number of surviving engrafted tumor cells varies by a factor of 10

In order to confirm the assumption that not all injected cells contribute to the formation of the initial tumor, but rather that a proportion of the injected cells die as the tumor becomes established in the connective tissue, an animal experiment was performed to examine the survival rate of the injected cells over a short period. We analyzed a total number of 8 mice in 4 different groups (days 1, 2, 4 and 8 after tumor cell injection, *n* = 2 each).

**Fig. 5:**
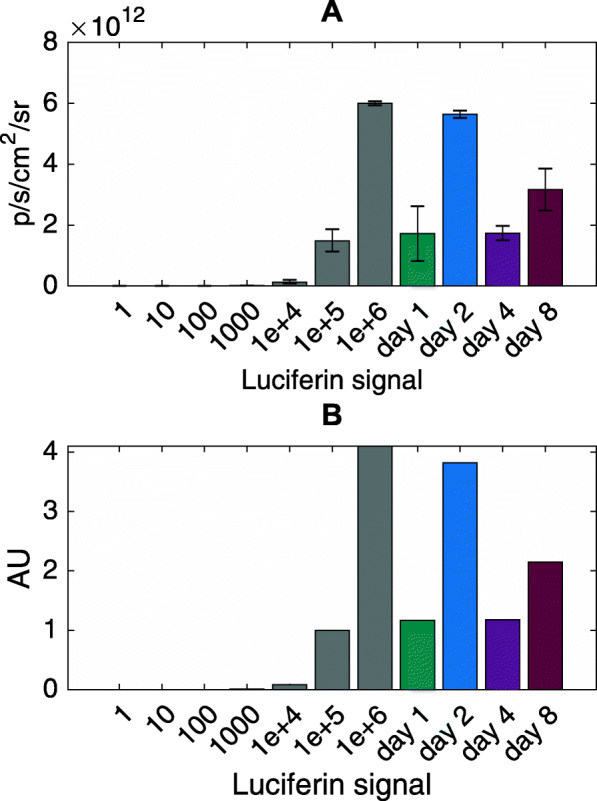
Bioluminescence signals from cultured tumor cells and initial tumors. The luciferin signal is depicted for different numbers of tumor cells (gray) and resected tumor nodules on days 1 (green), 2 (blue), 4 (violet) and 8 (purple) in photons per second per square centimeter per steradian (p/s/cm^2^/sr) in **a** and normalized to 10^5^ cells in **b**

The cell mass of ex vivo tumors and diluted cell culture control cells was detected by bioluminescence imaging (BLI) of a luciferin signal. Comparing the photon flux emitted from the tumor nodules on day 1 post injection (p.i.) with that emitted from control cells, the intensity from the tumor nodule was 10 times lower than the intensity of 1*10^6^ cells (i.e. the number of cells initially injected). On day 2 p.i. the photon flux from the tumor reached the same level as that of the injected cell number (1*10^6^ cells), while on day 4 p.i. it decreased again to the equivalent of approximately 1*10^5^ cells. On day 8 p.i. the photon flux from the tumor nodule corresponded to that of 5*10^5^ cells (approx. 50% of the number of injected cells) (Fig. 5ab).

The animals’ health status was monitored according to the FELASA guidelines during the experiment. No pathogens known to influence the experiments were detected.

## Discussion

### Complexity of parameter estimation methods

The present study was undertaken to analyze the best way to collect data on tumor growth in animal experimentation for mathematical modeling. Parameter estimation methods such as classical least-squares or maximum likelihood estimation attempt to approximate the unknown growth parameters using the underlying tumor data. Patmanidis et al. showed that the use of MLE methods provides better growth parameter results when the growth rate and carrying capacity are unknown than the classical least-squares approach with a fixed carrying capacity. If neither parameter is fixed, the least-squares approach can provide better results than MLE [43]. Therefore, we decided to use the least-squares approach, since neither of the growth parameters (growth rate and carrying capacity) were fixed during the evaluation process. Different algorithms can be used for the least-squares approach, such as the trust-region reflective, Levenberg–Marquardt or the Nelder–Mead algorithm, to estimate growth parameters based on tumor data. Benzekry et al. found no significant differences between the parameter results when comparing these three algorithms using self-generated synthetic data [21]. Therefore, we used the trust-region reflective least-squares algorithm implemented in MATLAB (*lsqcurvefit*) as the parameter estimation procedure, which is also the default algorithm in MATLAB. Overall, the complex curve fitting process depends on various criteria such as the initial settings (fixed or non-fixed parameters, boundary conditions or maximum iterations for the selected algorithm) or the quality of the tumor data, which in turn depends on the volume determination technique, such as the use of calipers, ultrasound or magnetic resonance imaging. Based on the results of this study, we conclude that the least-squares minimization approach including a fixed initial tumor volume condition is able to determine tumor growth parameters sufficiently well if the number of surviving cells has been correctly determined.

### Experimental methods need to be enhanced for appropriate mathematical description of tumor growth

Mathematical models are used to describe tumor growth in experimental and human data in order to better understand how to treat and predict the growth of cancer. A common challenge is the selection of an appropriate model that is robust enough to cope with inaccurate and missing data points. For these incomplete and error-prone datasets, fit metrics can help to select a function that best describes the data points. However, our data support the previous finding that forming conclusions relating to long-term growth is difficult [34]. Our findings also show that for proper determination of the growth parameters it is important to determine the surviving number of engrafted cells and to measure tumor size every day.

### Survival of engrafted tumor cells

The results of the animal experiment indicate that after s.c. injection 90% of the human tumor cells died and were not established in the mouse connective tissue. However, within one further day, the number of viable cells rapidly increased to the level of the initially injected cell count (on day 2 p.i.). The strong proliferation seen between days 1 and 2 p.i. may have been triggered by growth factors included in the culture medium that was injected with the tumor cells. On day 4 p.i. the viable tumor cell numbers at the injection site decreased again to about 10% of the injected cell count (comparable to the situation on day 1). We suggest that in this phase the injected growth factors have been consumed and the tumor cells are not well enough established in the connective tissue to be in contact with the vascular system, which is necessary for them to be adequately supplied with host growth factors. The increased tumor cell count on day 8 p.i. suggests that the tumor now has a functioning connection to the vascular system and further tumor growth can be expected.

### The absolute number of surviving engrafted tumor cells is difficult to assess during an experiment

**Fig. 6:**
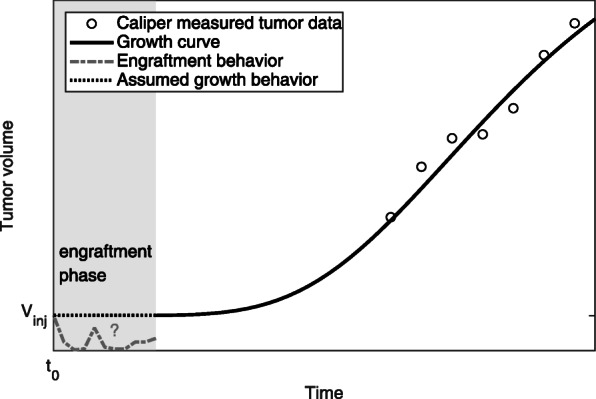
Schemata of the engraftment phase after injection of tumor cells. Typically, the number of injected cells (*V*_*inj*_) is used to perform nonlinear curve fitting, which leads to erroneous parameter results (black dotted line). Experimental data indicate that the first days of tumor nodule growth are not simple but more complex (gray dashed line)

We have highlighted the significant problem that knowing the correct number of surviving engrafted tumor cells is very important in order to improve parameter estimation results and describe actual tumor characteristics such as the aggressiveness (growth rate) of the tumor. Our data indicate that the number of surviving tumor cells is indeed smaller than the number of injected cells and does vary widely. However, in an ongoing experiment it is practically impossible to determine the number of surviving engrafted tumor cells during the first days after the injection using standard tumor volume determination techniques. With all standard methods for determining the tumor volume, including palpation, calipers, X-ray, ultrasound or magnetic resonance imaging, the tumor nodule is too small to be detected during the first days/weeks after primary engraftment of the injected tumor cells. Because of the experimental limitations, little is known about the course of events during this period (Fig. 6). This problem is further complicated by the fact that the number of tumor cells is calculated from the tumor volume and assumptions must be made about the structure of the tumor itself, such as the proportions of blood vessels, stroma cells and malignant cells. Further experimental research is required to improve understanding of the engraftment phase during experiments and to develop a novel technique to determine the number of surviving engrafted tumor cells in vivo.

### Preclinical implications

Our results indicate that the number of animals per group plays an important role in selecting a suitable growth function based on goodness of fit criteria. For the purpose of parameter estimation, at least 15 animals should be selected for an experiment. Research groups should also consider sample size calculation techniques to choose an appropriate number of animals depending on their research aims, for example power analysis [44, 45].

In terms of parameter estimation, the most accurate results were obtained with daily measurement to describe the underlying synthetic data including measurement errors. However, one should bear in mind that a high measurement frequency increases the burden on the animals. The measurement frequency should therefore be carefully selected depending on the aim of the experiment.

There is currently no optimum approach for choosing a more realistic value for the initial tumor volume in the fit. For the time being we propose a second control group to evaluate surviving engrafted tumor cells, similar to that used in this study. The large variation in the number of malignant cells that survive the first days following engraftment can lead to a misinterpretation of the aggressiveness or drug effects compared to a control group. Therefore, this information can then be used to improve the parametrization during the curve fitting procedure. In the long term, typical numbers of surviving tumor cells may be available for particular cell and mouse types. The definitive approach would be a novel experimental technique to determine the number of cells in the first days after injection. We speculate that optical methods would be a viable direction for further research.

## Conclusions

In this article, we have examined the mathematical evaluation process with respect to parameter estimation using nonlinear least-square curve fitting based on self-generated synthetic data that mimic caliper measured experimental xenograft mouse tumor data including measurement errors. Using this model we were able to determine that selection of a suitable growth function for experimental data requires at least 15 animals and that daily measurement provides the best basis for the evaluation of growth parameters to describe and predict the growth of cancer. Our study raises the question of the overall validity of parameter estimation results if the number of surviving engrafted tumor cells is not determined in experiments. We conclude that research into the development of engrafted cells during the first days is necessary for quantitative understanding of tumor growth in mouse models.

## Supplementary information

**Additional file 1: Table S1.** Parameter estimation of Gompertzian growth based on different data availability and growth behavior. Indicated are the mean values for each parameter. () = Minimum and maximum values for each parameter. {} = Parameter estimation bias (peb). [] = Coefficient of variation (CV). *Parameter *V*_0_ was set to 1 mm^3^ during the fitting procedure. True values: *V*_0_ = 1 mm^3^, *a* = 0.56 day^− 1^, *β* = 0.0719 day^− 1^.

**Additional file 2: Table S2.** Parameter estimation of Gompertzian growth based on different data availability and growth behavior (first measured tumor data point on day 14 with a size of about 150 mm3). Fitting were performed from day 14 to day 43, which corresponds to an initial tumor volume of about 150 mm^3^. Indicated are the mean values for each parameter. () = Minimum and maximum values for each parameter. {} = Parameter estimation bias (peb). [] = Coefficient of variation (CV). *Parameter V0 was set to 1 mm3 during the fitting procedure. True values: V0 = 1 mm^3^, a = 0.56 day^− 1^, β = 0.0719 day^− 1^.

**Additional file 3: Table S3.** Deviation between true and predicted tumor volumes using different time intervals between measurements based on 20 synthetic samples of Gompertzian growth (slow growth behavior). Indicated are the absolute mean values for each measurement frequency and the corresponding depth. () = 95% confidence interval. Parameter V0 was set to 1 mm3 during the fitting procedure. The mean RMSE was calculated based on the model fit in the period under consideration from day 23 to day 43. Growth parameters: V0 = 1 mm^3^, a = 0.4284 day^− 1^, β = 0.055 day^− 1^ (slow growth behavior).

**Additional file 4: Table S4.** Deviation between true and predicted tumor volumes using different time intervals between measurements based on 20 synthetic samples of Gompertzian growth (fast growth behavior). Indicated are the absolute mean values for each measurement frequency and the corresponding depth. () = 95% confidence interval. Parameter *V*_0_ was set to 1 mm^3^ during the fitting procedure. The mean RMSE was calculated based on the model fit in the period under consideration from day 23 to day 43. Growth parameters: V_0_ = 1 mm^3^, a = 0.7399 day^− 1^, β = 0.095 day^− 1^ (fast growth behavior).

**Additional file 5: File S1.** Gompertzian_growth_Metrics.xlsx. Fit metrics of all datasets based on Gompertzian growth.

**Additional file 6: Figure S1.** Effect of sample size on selection of most appropriate growth function for the underlying exponential data based on the lowest RMSE value. Each sample size (number of animals) was generated 10 times (synthetic dataset number) to mimic an exponential growth behavior including caliper measurement errors. For each growth function, Gompertz (Gomp), exponential (Exp), power (Pow) and logistic (Log), the RMSE value was determined for each individual sample within each dataset (e.g. dataset 2 and sample size 10). The relative frequency denotes how often each of the growth functions had the lowest RMSE value for an individual fit in each sample size group.

**Additional file 7: Figure S2.** Effect of sample size on selection of most appropriate growth function for the underlying power law data based on the lowest RMSE value. Each sample size (number of animals) was generated 10 times (synthetic dataset number) to mimic a power growth behavior including caliper measurement errors. For each growth function, Gompertz (Gomp), exponential (Exp), power (Pow) and logistic (Log), the RMSE value was determined for each individual sample within each dataset (e.g. dataset 2 and sample size 10). The relative frequency denotes how often each of the growth functions had the lowest RMSE value for an individual fit in each sample size group.

**Additional file 8: Figure S3.** Effect of sample size on selection of most appropriate growth function for the underlying logistic data based on the lowest RMSE value. Each sample size (number of animals) was generated 10 times (synthetic dataset number) to mimic a logistic growth behavior including caliper measurement errors. For each growth function, Gompertz (Gomp), exponential (Exp), power (Pow) and logistic (Log), the RMSE value was determined for each individual sample within each dataset (e.g. dataset 2 and sample size 10). The relative frequency denotes how often each of the growth functions had the lowest RMSE value for an individual fit in each sample size group.

**Additional file 9: Figure S4.** Box-plots of parameter estimation results based on different initial volume conditions (true initial tumor volume = 1 mm^3^). The initial volume *V*_0_ was fixed (x-axis) during the fitting procedure. Results are based on 20 synthetic data samples with different measuring frequencies (1, 2, 3 and 4 days between each time point).

**Additional file 10: Figure S5.** Box-plots of parameter estimation results based on different initial volume conditions (true initial tumor volume = 0.1 mm^3^). The initial volume *V*_0_ was fixed (x-axis) during the fitting procedure. Results are based on 20 synthetic data samples with different measuring frequencies (1, 2, 3 and 4 days between each time point).

**Additional file 11: Table S5.** Deviation between true and predicted tumor volumes using different time intervals between measurements based on 20 synthetic samples of Gompertzian growth. Indicated are the absolute mean values for each measurement frequency and the corresponding depth. () = 95% confidence interval. Parameter *V*_0_ was set to 0.1 mm^3^ during the fitting procedure. Growth parameters: V_0_ = 1 mm^3^, a = 0.56 day^− 1^, β = 0.0719 day^− 1^.

**Additional file 12: Table S6.** Deviation between true and predicted tumor volumes using different time intervals between measurements based on 20 synthetic samples of Gompertzian growth. Indicated are the absolute mean values for each measurement frequency and the corresponding depth. () = 95% confidence interval. Parameter *V*_0_ was set to 0.1 mm^3^ during the fitting procedure. Growth parameters: V_0_ = 0.1 mm^3^, a = 0.745 day^− 1^, β = 0.0719 day^− 1^.

**Additional file 13: Table S7.** Deviation between true and predicted tumor volumes using different time intervals between measurements based on 20 synthetic samples of Gompertzian growth. Indicated are the absolute mean values for each measurement frequency and the corresponding depth. () = 95% confidence interval. Parameter *V*_0_ was set to 1 mm^3^ during the fitting procedure. Growth parameters: V_0_ = 0.1 mm^3^, a = 0.745 day^− 1^, β = 0.0719 day^− 1^.

## Data Availability

The data supporting the conclusions of this article are included within the article and its additional files. MATLAB scripts used to generate synthetic data and all fit metric results to select a suitable growth function have been made available by the Open Science Framework (OSF) at the following DOI: 10.17605/OSF.IO/SRTD4.

## Abbreviations

MLE: Maximum likelihood estimation
RMSE: Root mean squared error
R^2^: R squared
AIC: Akaike information criterion
AICc: Akaike information criterion corrected
peb: Parameter estimation bias
CV: Coefficient of variation
BLI: Bioluminescence imaging
p.i.: Post injection
s.c.: Subcutaneously
