# Supplementary material for: The initial engraftment of tumor cells is critical for the future growth pattern: a mathematical study based on simulations and animal experiments
Source: BMC Cancer. 2020 Jun 5;20:524. doi: 10.1186/s12885-020-07015-9 (PMC7275472; doi:10.1186/s12885-020-07015-9)
Supplement: Supplementary file 1 — Additional file 1: Table S1. Parameter estimation of Gompertzian growth based on different data availability and growth behavior. Indicated are the mean values for each parameter. () = Minimum and maximum values for each parameter. {} = Parameter estimation bias (peb). [] = Coefficient of variation (CV). *Parameter V0 was set to 1 mm3 during the fitting procedure. True values: V0 = 1 mm3, a = 0.56 day− 1, β = 0.0719 day− 1. [file 12885_2020_7015_MOESM1_ESM.docx]

Table S1: Parameter estimation of Gompertzian growth based on different data availability and growth behavior.

| **Measuring frequency** | **V_0_ [mm^3^]** | **a [day^−1^]** | **β [day^−1^]** | **a* [day^−1^]** | **β* [day^−1^]** |
| --- | --- | --- | --- | --- | --- |
| Every day | 3.61 (0.01–9.48) {2.6141} [86.8475] | 0.52 (0.3–1.19) {−0.0788} [44.293] | 0.0664 (0.0524–0.0952) {−0.0765} [18.7014] | 0.5541 (0.5374–0.5848) {−0.0104} [2.0377] | 0.0709 (0.0681–0.0758)  {−0.0143} [2.624] |
| Every 2 days | 3.28 (0–12.25) {2.285} [112.9065] | 0.55 (0.28–1.24) {−0.0151} [46.5235] | 0.0681 (0.0499–0.0937) {−0.0528} [19.2275] | 0.5541 (0.527–0.583) {−0.0105} [2.7212] | 0.0708 (0.0664–0.0754)  {−0.0153} [3.4499] |
| Every 3 days | 5.11 (0–27.48) {4.1113} [164.8952] | 0.69 (0.2–1.55) {0.229} [57.3469] | 0.0716 (0.0376–0.1031) {−0.0035} [27.685] | 0.5513 (0.521–0.5918) {−0.0156} [3.1826] | 0.0702 (0.0655–0.0769)  {−0.0231} [4.0617] |
| Every 4 days | 3.83 (0–34.81) {2.828} [203.2788] | 0.62 (0.18–1.4) {0.0988} [51.8203] | 0.0698 (0.0345–0.1006) {−0.0294} [22.7016] | 0.5495 (0.5198–0.5806) {−0.0188} [3.2408] | 0.0699 (0.065–0.0751)  {−0.0271} [4.1432] |

Indicated are the mean values for each parameter. ( ) = Minimum and maximum values for each parameter. { } = Parameter estimation bias (peb). [ ] = Coefficient of variation (CV). *Parameter V_0_ was set to 1 mm^3^ during the fitting procedure. True values: V_0_ = 1 mm^3^, a = 0.56 day^−1^, β = 0.0719 day^−1^.
