# Supplementary material for: The initial engraftment of tumor cells is critical for the future growth pattern: a mathematical study based on simulations and animal experiments
Source: BMC Cancer. 2020 Jun 5;20:524. doi: 10.1186/s12885-020-07015-9 (PMC7275472; doi:10.1186/s12885-020-07015-9)
Supplement: Supplementary file 2 — Additional file 2: Table S2. Parameter estimation of Gompertzian growth based on different data availability and growth behavior (first measured tumor data point on day 14 with a size of about 150 mm3). Fitting were performed from day 14 to day 43, which corresponds to an initial tumor volume of about 150 mm3. Indicated are the mean values for each parameter. () = Minimum and maximum values for each parameter. {} = Parameter estimation bias (peb). [] = Coefficient of variation (CV). *Parameter V0 was set to 1 mm3 during the fitting procedure. True values: V0 = 1 mm3, a = 0.56 day− 1, β = 0.0719 day− 1. [file 12885_2020_7015_MOESM2_ESM.docx]

Table S2: Parameter estimation of Gompertzian growth based on different data availability and growth behavior (first measured tumor data point on day 14 with a size of about 150 mm3).

| **Measuring frequency** | **V_0_ [mm^3^]** | **a [day^−1^]** | **β [day^−1^]** | **a* [day^−1^]** | **β* [day^−1^]** |
| --- | --- | --- | --- | --- | --- |
| Every day | 1.2 (0.04 - 2.66) {0.2004} [64.5324] | 0.59 (0.43 - 1.02) {0.0522} [25.5873] | 0.0729 (0.0617 - 0.0947) {0.014} [11.4345] | 0.56 (0.54 - 0.59) {0.0038} [1.8782] | 0.0722 (0.0692 - 0.0763) {0.0039} [2.398] |
| Every 2 days | 1.45 (0 - 4.61) {0.4524} [90.2391] | 0.62 (0.38 - 1.42) {0.0988} [39.5581] | 0.0730 (0.0586 - 0.1073) {0.0148} [16.7192] | 0.56 (0.53 - 0.61) {-0.0011} [3.0246] | 0.0717 (0.0676 - 0.0792) {-0.0026} [3.8242] |
| Every 3 days | 1.91 (0.05 - 7.75) {0.911} [122.7095] | 0.59 (0.31 - 0.96) {0.0496} [31.4317] | 0.0720 (0.0501 - 0.0911) {0.0017} [17.0288] | 0.56 (0.53 - 0.58) {0.0018} [3.2715] | 0.0721 (0.067 - 0.0754) {0.0021} [4.1131] |
| Every 4 days | 2.47 (0 - 8.41) {1.4667} [117.9455] | 0.63 (0.3 - 1.41) {0.1279} [48.9694] | 0.0723 (0.0495 - 0.1074) {0.0058} [23.0187] | 0.56 (0.52 - 0.61) {-0.0023} [4.1093] | 0.0717 (0.0659 - 0.0807) {-0.0034} [5.1896] |

Fitting were performed from day 14 to day 43, which corresponds to an initial tumor volume of about 150 mm^3^. Indicated are the mean values for each parameter. ( ) = Minimum and maximum values for each parameter. { } = Parameter estimation bias (peb). [ ] = Coefficient of variation (CV). *Parameter V_0_ was set to 1 mm^3^ during the fitting procedure. True values: V_0_ = 1 mm^3^, a = 0.56 day^−1^, β = 0.0719 day^−1^.
