# Supplementary material for: The initial engraftment of tumor cells is critical for the future growth pattern: a mathematical study based on simulations and animal experiments
Source: BMC Cancer. 2020 Jun 5;20:524. doi: 10.1186/s12885-020-07015-9 (PMC7275472; doi:10.1186/s12885-020-07015-9)
Supplement: Supplementary file 3 — Additional file 3: Table S3. Deviation between true and predicted tumor volumes using different time intervals between measurements based on 20 synthetic samples of Gompertzian growth (slow growth behavior). Indicated are the absolute mean values for each measurement frequency and the corresponding depth. () = 95% confidence interval. Parameter V0 was set to 1 mm3 during the fitting procedure. The mean RMSE was calculated based on the model fit in the period under consideration from day 23 to day 43. Growth parameters: V0 = 1 mm3, a = 0.4284 day− 1, β = 0.055 day− 1 (slow growth behavior). [file 12885_2020_7015_MOESM3_ESM.docx]

**Table S3: Deviation between true and predicted tumor volumes using different time intervals between measurements based on 20 synthetic samples of Gompertzian growth (slow growth behavior).**

| **Measuring frequency (days between measurements)** | $\bar{\boldsymbol{RMSE}}$ | **Absolute mean deviation from true volume at depth d [mm^3^]** | | | |
| --- | --- | --- | --- | --- | --- |
|  |  | **1** | **3** | **5** | **10** |
| 1 | 68.26 | 39.35 (29.28 ; 49.42) | 47.98 (35.15 ; 60.81) | 56.94 (41.14 ; 72.74) | 80 (56.21 ; 103.79) |
| 2 | 71.67 | 47.3 (31.73 ; 62.87) | 60.48 (41.28 ; 79.68) | 74.63 (51.58 ; 97.68) | 111.85 (78.09 ; 145.61) |
| 3 | 73.65 | 52.26 (34.28 ; 70.24) | 64.88 (41.33 ; 88.43) | 78.63 (49.16 ; 108.1) | 116.62 (71.63 ; 161.61) |
| 4 | 75.80 | 58.67 (43.81 ; 73.53) | 74.52 (55.13 ; 93.91) | 91.26 (66.56 ; 115.96) | 135.34 (95.21 ; 175.47) |

Indicated are the absolute mean values for each measurement frequency and the corresponding depth. ( ) = 95% confidence interval. Parameter *V*_0_ was set to 1 mm^3^ during the fitting procedure. The mean RMSE was calculated based on the model fit in the period under consideration from day 23 to day 43. Growth parameters: V_0_ = 1 mm^3^, a = 0.4284 day^−1^, β = 0.055 day^−1^ (slow growth behavior).
