# Supplementary material for: The initial engraftment of tumor cells is critical for the future growth pattern: a mathematical study based on simulations and animal experiments
Source: BMC Cancer. 2020 Jun 5;20:524. doi: 10.1186/s12885-020-07015-9 (PMC7275472; doi:10.1186/s12885-020-07015-9)
Supplement: Supplementary file 4 — Additional file 4: Table S4. Deviation between true and predicted tumor volumes using different time intervals between measurements based on 20 synthetic samples of Gompertzian growth (fast growth behavior). Indicated are the absolute mean values for each measurement frequency and the corresponding depth. () = 95% confidence interval. Parameter V0 was set to 1 mm3 during the fitting procedure. The mean RMSE was calculated based on the model fit in the period under consideration from day 23 to day 43. Growth parameters: V0 = 1 mm3, a = 0.7399 day− 1, β = 0.095 day− 1 (fast growth behavior). [file 12885_2020_7015_MOESM4_ESM.docx]

**Table S4: Deviation between true and predicted tumor volumes using different time intervals between measurements based on 20 synthetic samples of Gompertzian growth (fast growth behavior).**

| **Measuring frequency (days between measurements)** | $\bar{\boldsymbol{RMSE}}$ | **Absolute mean deviation from true volume at depth d [mm^3^]** | | | |
| --- | --- | --- | --- | --- | --- |
|  |  | **1** | **3** | **5** | **10** |
| 1 | 114.74 | 53.65 (39.31 ; 67.99) | 58.87 (43.36 ; 74.38) | 63.53 (46.92 ; 80.14) | 72.92 (53.91 ; 91.93) |
| 2 | 118.44 | 63.71 (49.86 ; 77.56) | 68.88 (53.52 ; 84.24) | 73.49 (56.67 ; 90.31) | 82.93 (63.06 ; 102.8) |
| 3 | 120.11 | 62.97 (45.26 ; 80.68) | 70.36 (51.47 ; 89.25) | 76.96 (56.88 ; 97.04) | 90.73 (68.31 ; 113.15) |
| 4 | 120.27 | 83.66 (60.9 ; 106.42) | 89.78 (64.87 ; 114.69) | 95.41 (68.63 ; 122.19) | 107.09 (76.59 ; 137.59) |

Indicated are the absolute mean values for each measurement frequency and the corresponding depth. ( ) = 95% confidence interval. Parameter *V*_0_ was set to 1 mm^3^ during the fitting procedure. The mean RMSE was calculated based on the model fit in the period under consideration from day 23 to day 43. Growth parameters: V_0_ = 1 mm^3^, a = 0.7399 day^−1^, β = 0.095 day^−1^ (fast growth behavior).
