# Supplementary figures and images for: The initial engraftment of tumor cells is critical for the future growth pattern: a mathematical study based on simulations and animal experiments
Source: BMC Cancer. 2020 Jun 5;20:524. doi: 10.1186/s12885-020-07015-9 (PMC7275472; doi:10.1186/s12885-020-07015-9)

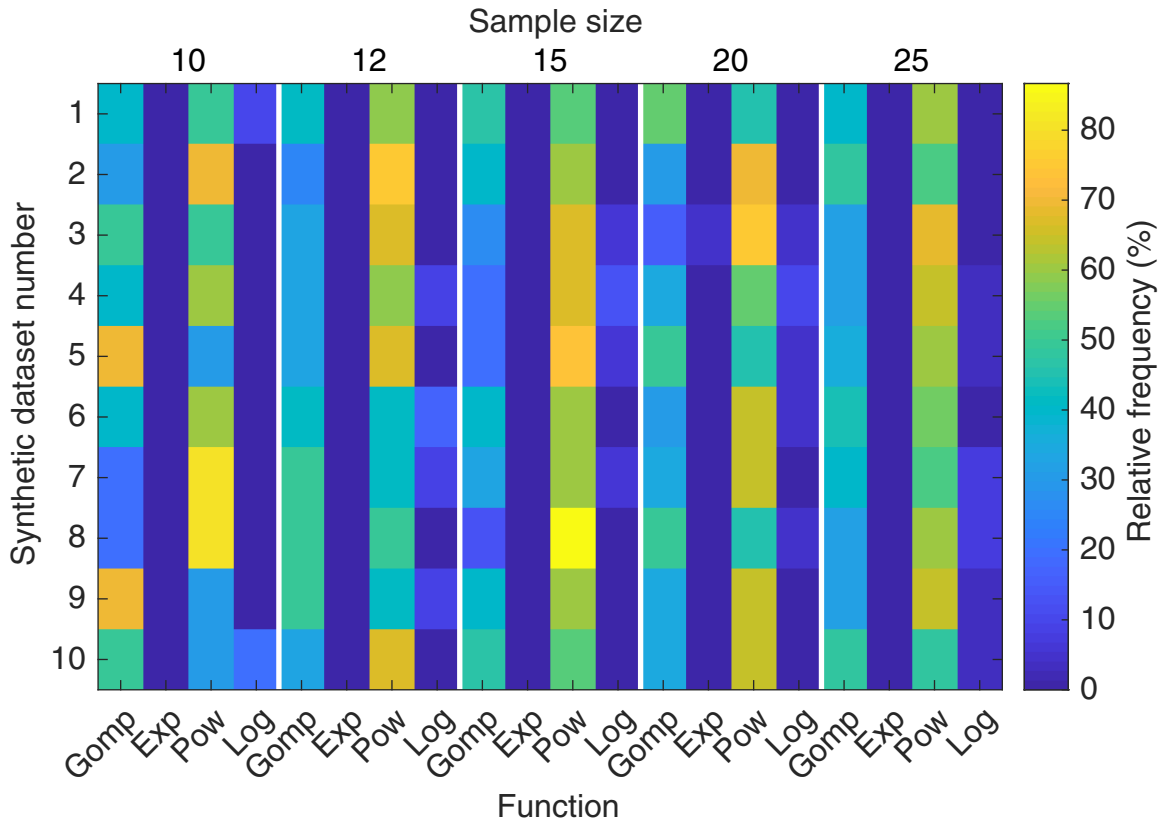

Supplement: Supplementary file 6 — Additional file 6: Figure S1. Effect of sample size on selection of most appropriate growth function for the underlying exponential data based on the lowest RMSE value. Each sample size (number of animals) was generated 10 times (synthetic dataset number) to mimic an exponential growth behavior including caliper measurement errors. For each growth function, Gompertz (Gomp), exponential (Exp), power (Pow) and logistic (Log), the RMSE value was determined for each individual sample within each dataset (e.g. dataset 2 and sample size 10). The relative frequency denotes how often each of the growth functions had the lowest RMSE value for an individual fit in each sample size group. [file 12885_2020_7015_MOESM6_ESM.pdf]

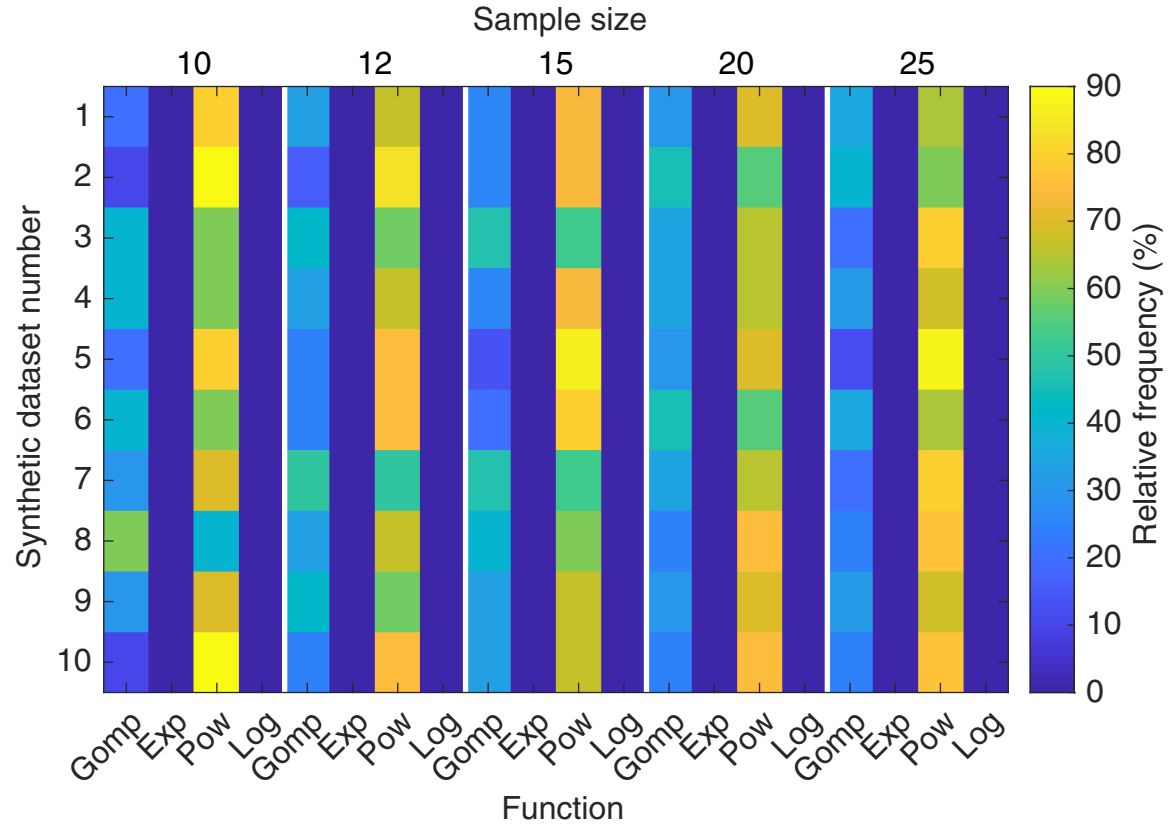

Supplement: Supplementary file 7 — Additional file 7: Figure S2. Effect of sample size on selection of most appropriate growth function for the underlying power law data based on the lowest RMSE value. Each sample size (number of animals) was generated 10 times (synthetic dataset number) to mimic a power growth behavior including caliper measurement errors. For each growth function, Gompertz (Gomp), exponential (Exp), power (Pow) and logistic (Log), the RMSE value was determined for each individual sample within each dataset (e.g. dataset 2 and sample size 10). The relative frequency denotes how often each of the growth functions had the lowest RMSE value for an individual fit in each sample size group. [file 12885_2020_7015_MOESM7_ESM.pdf]

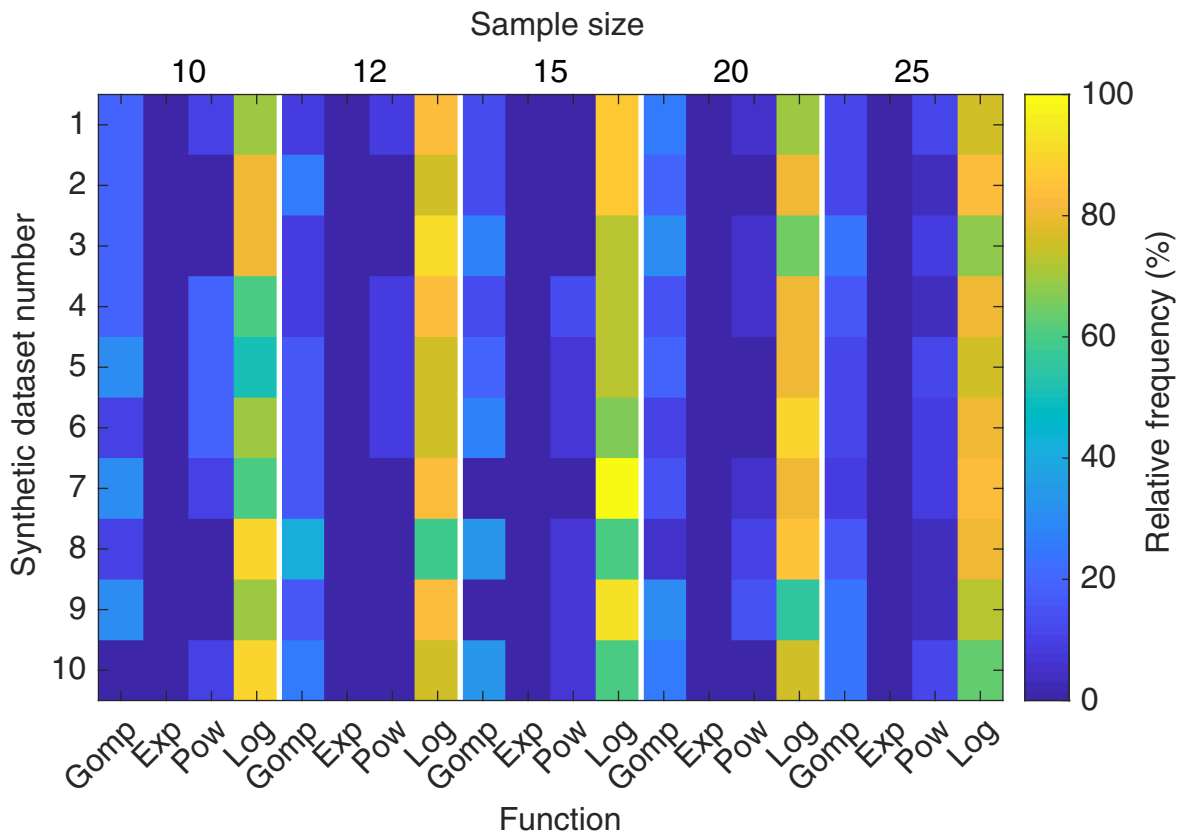

Supplement: Supplementary file 8 — Additional file 8: Figure S3. Effect of sample size on selection of most appropriate growth function for the underlying logistic data based on the lowest RMSE value. Each sample size (number of animals) was generated 10 times (synthetic dataset number) to mimic a logistic growth behavior including caliper measurement errors. For each growth function, Gompertz (Gomp), exponential (Exp), power (Pow) and logistic (Log), the RMSE value was determined for each individual sample within each dataset (e.g. dataset 2 and sample size 10). The relative frequency denotes how often each of the growth functions had the lowest RMSE value for an individual fit in each sample size group. [file 12885_2020_7015_MOESM8_ESM.pdf]

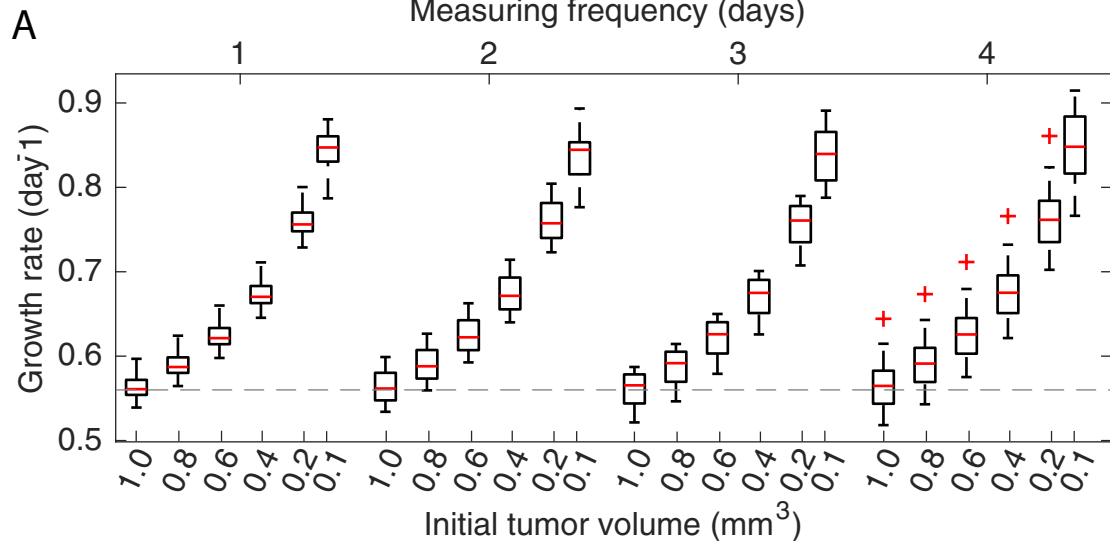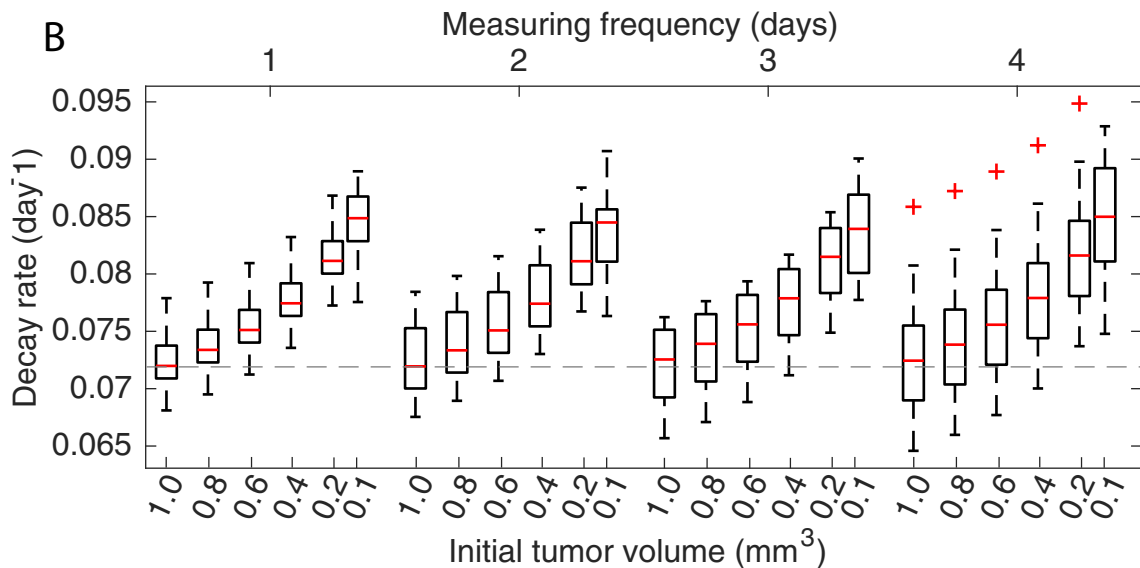

Supplement: Supplementary file 9 — Additional file 9: Figure S4. Box-plots of parameter estimation results based on different initial volume conditions (true initial tumor volume = 1 mm3). The initial volume V0 was fixed (x-axis) during the fitting procedure. Results are based on 20 synthetic data samples with different measuring frequencies (1, 2, 3 and 4 days between each time point). [file 12885_2020_7015_MOESM9_ESM.pdf]

Measuring frequency (days)

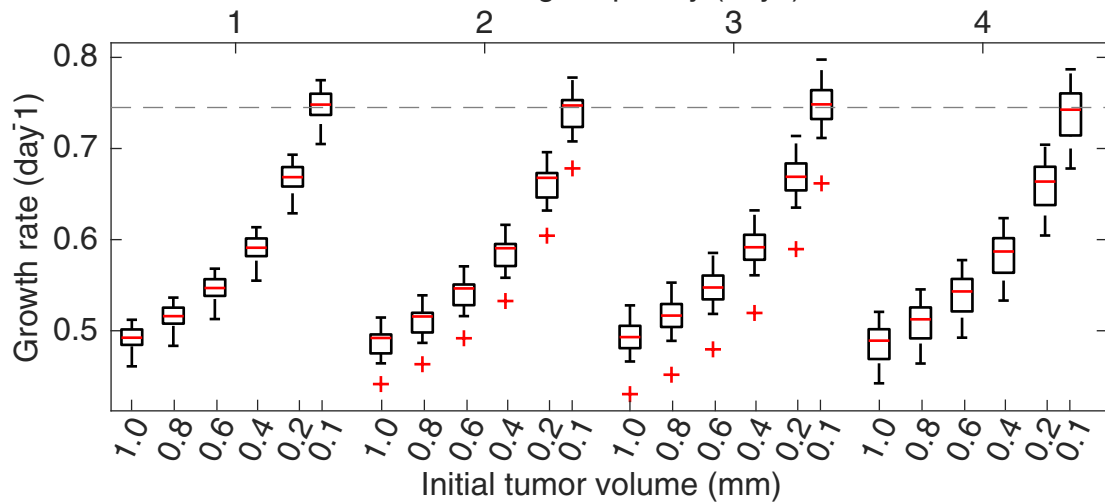

Measuring frequency (days)

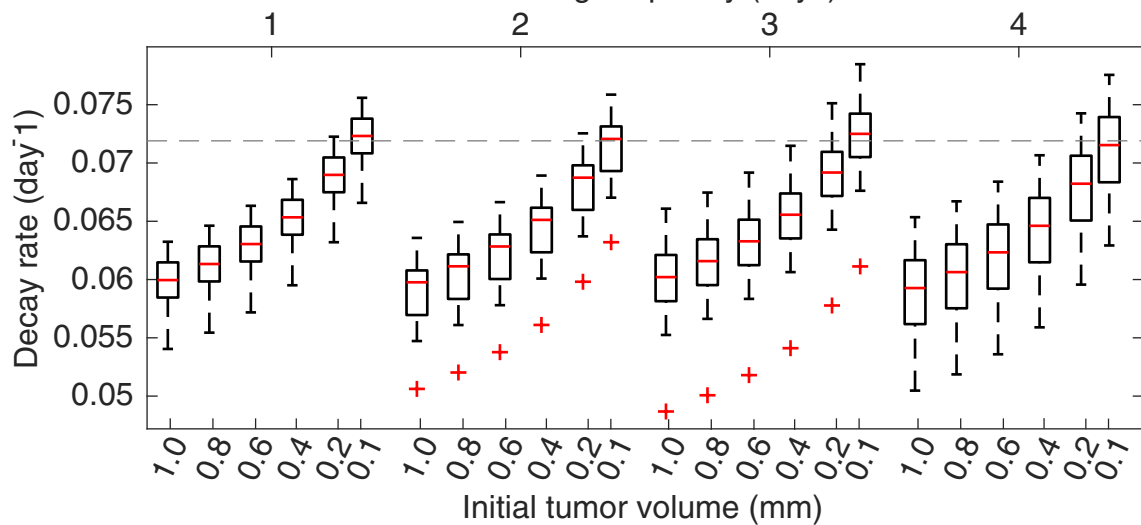

Supplement: Supplementary file 10 — Additional file 10: Figure S5. Box-plots of parameter estimation results based on different initial volume conditions (true initial tumor volume = 0.1 mm3). The initial volume V0 was fixed (x-axis) during the fitting procedure. Results are based on 20 synthetic data samples with different measuring frequencies (1, 2, 3 and 4 days between each time point). [file 12885_2020_7015_MOESM10_ESM.pdf]
