# Supplementary material for: The initial engraftment of tumor cells is critical for the future growth pattern: a mathematical study based on simulations and animal experiments
Source: BMC Cancer. 2020 Jun 5;20:524. doi: 10.1186/s12885-020-07015-9 (PMC7275472; doi:10.1186/s12885-020-07015-9)
Supplement: Supplementary file 12 — Additional file 12: Table S6. Deviation between true and predicted tumor volumes using different time intervals between measurements based on 20 synthetic samples of Gompertzian growth. Indicated are the absolute mean values for each measurement frequency and the corresponding depth. () = 95% confidence interval. Parameter V0 was set to 0.1 mm3 during the fitting procedure. Growth parameters: V0 = 0.1 mm3, a = 0.745 day− 1, β = 0.0719 day− 1. [file 12885_2020_7015_MOESM12_ESM.docx]

**Table S6: Deviation between true and predicted tumor volumes using different time intervals between measurements based on 20 synthetic samples of Gompertzian growth.**

| **Measuring frequency (days between measurements)** | **Absolute mean deviation from true volume at depth d [mm^3^]** | | | |
| --- | --- | --- | --- | --- |
|  | **1** | **3** | **5** | **10** |
| 1 | 57.01 (35.6 ; 78.42) | 67.7 (42.41 ; 92.99) | 78.22 (49.04 ; 107.4) | 102.84 (64.33 ; 141.35) |
| 2 | 79.05 (50.11 ; 107.99) | 91.27 (55.34 ; 127.2) | 103.26 (60.04 ; 146.48) | 131.71 (70.39 ; 193.03) |
| 3 | 86.66 (50.97 ; 122.35) | 102.07 (57.69 ; 146.45) | 117.29 (63.99 ; 170.59) | 154.21 (79.15 ; 229.27) |
| 4 | 96.14 (59.67 ; 132.61) | 112.15 (67.69 ; 156.61) | 127.89 (74.99 ; 180.79) | 166.07 (92.33 ; 239.81) |

Indicated are the absolute mean values for each measurement frequency and the corresponding depth. ( ) = 95% confidence interval. Parameter *V*_0_ was set to 0.1 mm^3^ during the fitting procedure. Growth parameters: V_0_ = 0.1 mm^3^, a = 0.745 day^−1^, β = 0.0719 day^−1^.
